# Distribution of endogenous gammaretroviruses and variants of the *Fv1* restriction gene in individual mouse strains and strain subgroups

**DOI:** 10.1371/journal.pone.0219576

**Published:** 2019-07-10

**Authors:** Matthew Skorski, Devinka Bamunusinghe, Qingping Liu, Esther Shaffer, Christine A. Kozak

**Affiliations:** Laboratory of Molecular Microbiology, National Institute of Allergy and Infectious Diseases, Bethesda, Maryland, United States of America; University Hospital Tuebingen, GERMANY

## Abstract

Inbred laboratory mouse strains carry endogenous retroviruses (ERVs) classed as ecotropic, xenotropic or polytropic mouse leukemia viruses (E-, X- or P-MLVs). Some of these MLV ERVs produce infectious virus and/or contribute to the generation of intersubgroup recombinants. Analyses of selected mouse strains have linked the appearance of MLVs and virus-induced disease to the strain complement of MLV E-ERVs and to host genes that restrict MLVs, particularly *Fv1*. Here we screened inbred strain DNAs and genome assemblies to describe the distribution patterns of 45 MLV ERVs and *Fv1* alleles in 58 classical inbred strains grouped in two ways: by common ancestry to describe ERV inheritance patterns, and by incidence of MLV-associated lymphomagenesis. Each strain carries a unique set of ERVs, and individual ERVs are present in 5–96% of the strains, often showing lineage-specific distributions. Two ERVs are alternatively present as full-length proviruses or solo long terminal repeats. High disease incidence strains carry the permissive *Fv1*^*n*^ allele, tested strains have highly expressed E-ERVs and most have the *Bxv1* X-ERV; these three features are not present together in any low-moderate disease strain. The P-ERVs previously implicated in P-MLV generation are not preferentially found in high leukemia strains, but the three *Fv1* alleles that restrict inbred strain E-MLVs are found only in low-moderate leukemia strains. This dataset helps define the genetic basis of strain differences in spontaneous lymphomagenesis, describes the distribution of MLV ERVs in strains with shared ancestry, and should help annotate sequenced strain genomes for these insertionally polymorphic and functionally important proviruses.

## Introduction

The multiple inbred strains of laboratory mice carry three host range subgroups of MLVs (reviewed in [1]). MLVs with ecotropic host range (E-MLVs) infect only rodent cells, while the various xenotropic and polytropic MLVs (X-, P-MLVs, collectively X/P-MLVs) infect different subsets of mouse taxa and other mammalian species [2, 3]. The E-MLVs all use the CAT1 receptor [4], and the X/P-MLVs all use the functionally polymorphic XPR1 receptor [5].

These three host range subgroups of MLVs are found as infectious viruses and as endogenous retroviruses (ERVs), which are DNA copies that integrated into the germline during past virus infections and were passed to subsequent generations. Individual ERVs can be present or absent in the various mouse strains. Over 30 distinct E-MLV ERVs (E-ERVs, termed *Emvs*) are found in laboratory mice, and individual strains can carry up to six *Emv*s [6], many of which are capable of producing infectious virus [1]. Laboratory strains also carry X-ERVs (*Xmvs*) [7], some of which can produce virus [8], and two subclasses of P-ERVs, the polytropic murine viruses (*Pmvs*) and modified polytropic murine viruses (*Mpmvs*) [9], none of which have infectious virus counterparts, although they can contribute to the generation of intersubgroup recombinant viruses that have the distinctive P-MLV host range [10–13].

The classical inbred strains were derived from mice provided to research laboratories at the turn of the last century by fancy mouse hobbyists. These fancy mice, bred for centuries as pets and for show, were produced by interbreeding wild house mice of three *M*. *musculus* subspecies (*castaneus*, *musculus*, *domesticus*) [14]. These classical laboratory strains and their fancy mouse progenitors have been shown to be intersubspecific mosaics [15, 16].

All 3 *M*. *musculus* subspecies carry MLV ERVs, but the distribution of ERV subtypes in wild house mice is segregated by geography and subspecies [17]. *M*. *m*. *domesticus* of Western Europe carries only P-MLV ERVs, whereas E-MLV ERVs and X-MLV ERVs are found only in *M*. *m*. *castaneus* and *M*. *m*. *musculus* in eastern Europe and Asia, and in their naturally occurring Japanese hybrid, *M*. *m*. *molossinus* [17–19]. The fancy mouse intersubspecies hybrids and the inbred laboratory strains ultimately acquired all three MLV subtypes, often in the absence of the protective antiviral host factors found in virus-infected wild mouse populations [20]. As a result, fancy mice were afflicted with naturally occurring tumors and were formally studied as mammalian models of cancer as far back as the turn of the last century [21].

Inbred mouse strains have been especially useful in genetic studies and as models of human disease. After their introduction into the laboratory, mice showing a high incidence of spontaneous disease were deliberately inbred to generate, for example, the “high leukemia” strains such as AKR, as well as other models of human disease including strains having high incidence of mammary tumors (C3H) [22], lupus-like autoimmune disorders (NZB) [23], accelerated senescence (SAMP) [24] and diabetes (NOD) [25]. Retroviruses were investigated as causative agents in many of these naturally occurring diseases [26] and MLVs have a well-documented etiological association with the induction of lymphomas [27]. Strain differences in lymphoma incidence have long been attributed to the presence of MLVs and host factors that affect their replication, but these factors have been evaluated in only a selected subset of the laboratory strains, largely through classical genetic crosses [28].

We previously developed oligonucleotide primer sets specific for 43 X/P-ERVs in the sequenced C57BL/6 genome (here termed B6) to trace individual MLV ERVs to their wild mouse progenitors [29]. Here we expanded our analysis of the inbred strains to formally detail the distribution patterns of 45 *Pmvs*, *Mpmvs*, and *Xmvs* in 58 inbred strains and to screen the recently assembled sequenced genomes of 11 of these strains [30] for expected as well as novel MLV ERVs. We describe the ERV content of strains subgrouped in two ways: first, on the basis of their common origins, known breeding histories and genetic differences [15], and second, on the basis of their documented incidence of hematopoietic neoplasms. We show that all 45 of the X/P-ERVs found in the B6 genome are found in other strains, but that each strain has a unique complement of these ERVs, information that should be useful in annotating these repetitive and insertionally polymorphic ERVs in the sequenced genomes of the various mouse strains and species of wild mice. The presence of individual ERVs previously implicated in the generation of P-MLV recombinants [13] was compared with strain disease profiles as was the distribution of alleles of the mouse *Fv1* gene which has a major role in restricting MLV spread [31]. High leukemia incidence was linked to the presence of specific expressed E- and X-ERVs and to the permissive *Fv1*^*n*^ allele; low leukemia strains generally lack active MLVs and/or carry the restrictive *Fv1* alleles.

## Materials and methods

### Mouse DNAs

DNAs from 41 strains were purchased as DNAs or isolated from mice obtained from The Jackson Laboratory (Bar Harbor, ME). DNAs from ten senescence-accelerated (SAM) mice were prepared from tissues obtained from Richard Carp (NY State Institute for Basic Research in Developmental Disabilities, NYC, NY). DNAs from six strains (F/St, SIM.R, SIM, DBA/2N, AKR/N and NFS/N) were isolated from livers of mice maintained in our laboratory. The two SIM strains were originally obtained from D. Axelrad [32].

### Virus restriction by Fv1

Susceptibility to Fv1-sensitive MLVs was assessed using the UV-XC overlay assay as described previously [33]. The tested cells included NIH 3T3 (*Fv1*^*n*^), BALB 3T3 (*Fv1*^*b*^) and fully permissive SC-1 cells [34] originally obtained from Dr. J. Hartley (National Institute of Allergy and Infectious Diseases, Bethesda, MD) and fibroblast lines developed in our laboratory from 129 (*Fv1*^*nr*^) and DBA (*Fv1*^*d*^) mouse embryos. Prototype viruses sensitive or resistant to *Fv1* alleles included AKV (N-tropic), WN1802B (B-tropic), AKR-L1 (NR-tropic) and Moloney MLV (MoMLV, NB-tropic), all of which were originally obtained from Dr. J. Hartley.

### Distribution of MLV ERVs in inbred strains

Primer pairs were previously designed for 19 *Pmvs*, 13 *Xmvs* and 12 *Mpmvs* to generate diagnostic 3’ and 5’ cell-virus junction fragments and to identify the empty pre-integration sites and possible solo LTRs [29, 35]. Primers flanking an additional *Xmv*, *Xmv16*, were: Xmv16F1, 5’-CTCATCTCTGGGTCTTGGTCC; Xmv16R2, 5’-CTCAGTCTACATTCAGCCTTCC.

Strains not previously assessed for *Emvs* were typed by PCR using AKV E-MLV specific *env* primers: EcoF: 5’-CGAGAAACGGTGTGGGCAATAAC and EcoR: 5’-GGTTGCCTGGTCTGAGGTTAGATTGTTGCTTACTGTGATG.

40 of the 58 strains had been characterized with single nucleotide pairs and variable intensity oligonucleotides to produce a high-density genotyped dataset [36]. The resulting high-resolution genetic maps were used to define phylogenetic relationships and subspecific origins [37]. We used this dataset, available through the Mouse Phylogeny Viewer (MPV) at the University of North Carolina (http://msub.csbio.unc.edu) [36, 38], to examine the genomic segments surrounding each ERV integration site across the 40 strains, and to identify their wild mouse subspecific origins and their association with shared haplotype segments. This analysis used ERV chromosome coordinates from the NCBI37/mm9 reference assembly identified by BLAT searches [39] for each of the 45 MLV ERVs using the UCSC Genome browser (http://genome.ucsc.edu).

Draft genome sequences for 11 of the 58 strains have been produced [30]. All 11 were screened for MLV proviruses related to known and novel *Emvs*, *Pmvs*, *Mpmvs*, and 4 subtypes of *Xmvs*, and for sequences that flank the B6 MLV ERVs. Screens used BLASTn [39] at NCBI or the BLAST/BLAT tool on the Ensembl website [40] (https://useast.ensembl.org/Mus_musculus/Tools/Blast?db=core).

### Sequencing of selected insertion sites and *Fv1*

Pre-integration ERV sites with larger than expected sizes were cloned into pCR2.1-TOPO (Invitrogen, Carlsbad, CA) and sequenced.

*Fv1* was amplified from mouse genomic DNAs using primers from *Fv1*^*b*^ (GenBank No. X97719): 5’-AGATGAATTTCCCACGTGC, 5’-CATCTATACTATCTTGGTGAG. These primers generate an *Fv1*^*b*^ product of 2.5 kb and products of 1.3 kb from strains carrying *Fv1*^*n*^, *Fv1*^*nr*^ and *Fv1*^*d*^. *Fv1* products from 43 inbred strains were cloned into pCR2.1-TOPO before sequencing.

Sequences were deposited in GenBank for 3 strain-specific insertion sites carrying solo LTRs (MH479985-7) and for *Fv1*^*d*^ (MH479988). Identical sequences for *Fv1*^*d*^ were identified in DNAs from substrains DBA/2J and DBA/2N, and in strains NZO/HlLtJ, MRL/MpJ, TALLYHO/JnJ, SOD1/EiJ and LG/J.

### Ethics statement

This study was carried out in strict accordance with the recommendations in the Guide for the Care and Use of Laboratory Animals of the National Institutes of Health and procedures were in accordance with the guidelines of the Committee of the Care and Use of Laboratory Animals under the NIAID-approved animal study protocol LMM1, which was approved by the Institutional Animal Care and Use Committee. Eight of the 58 mouse DNAs and two mouse embryo fibroblast lines were produced under this protocol from mice euthanized by CO_2_ inhalation in accordance with Animal Research Advisory Committee guidelines. Three mouse DNAs were isolated from mouse livers in the early 1980s, prior to the establishment of any Animal Care and Use Committee and the rest of the DNAs were purchased or isolated from livers provided by outside sources named above.

## Results and discussion

### Distribution of X/P-MLV ERVs in 58 laboratory strains

**Fig 1:**
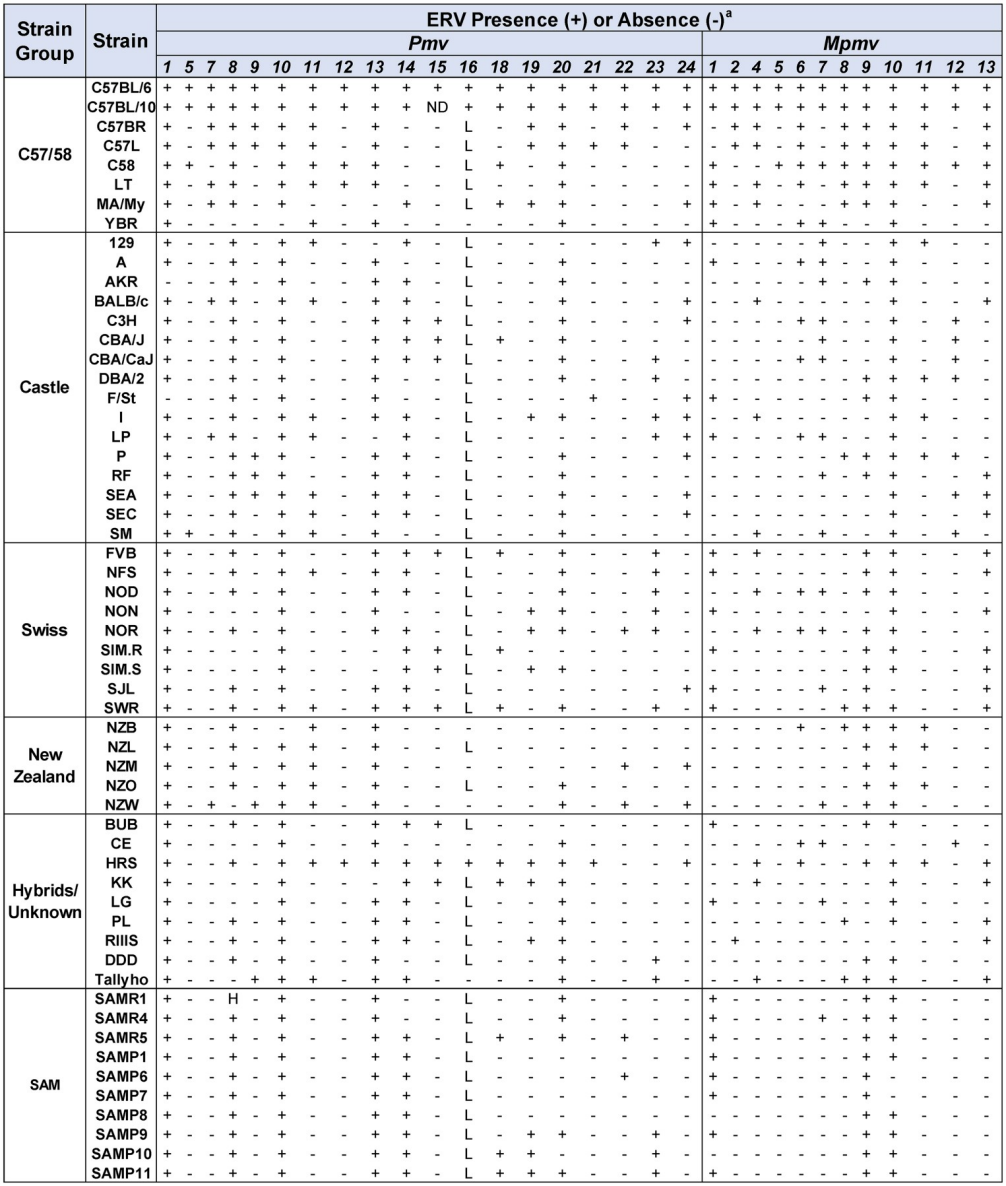
Classical mouse strains typed for 31 P-MLVs and grouped according to known breeding history. Substrains AKR/N and AKR/J, and substrains C57BL/6 and C57BL/10 carry identical ERVs, whereas the two CBA substrains differ for six ERVs likely due to genetic contamination [37]. The SIM.R *Fv1*^*b*^ congenic differs from its SIM progenitor in the presence of 6 ERVs found in the *Fv1*^*b*^ donor strain, three of which are linked to *Fv1*. ^a^+, presence; -, absence; L, solo LTR; H, heterozygous; ND, not done.

**Fig 2:**
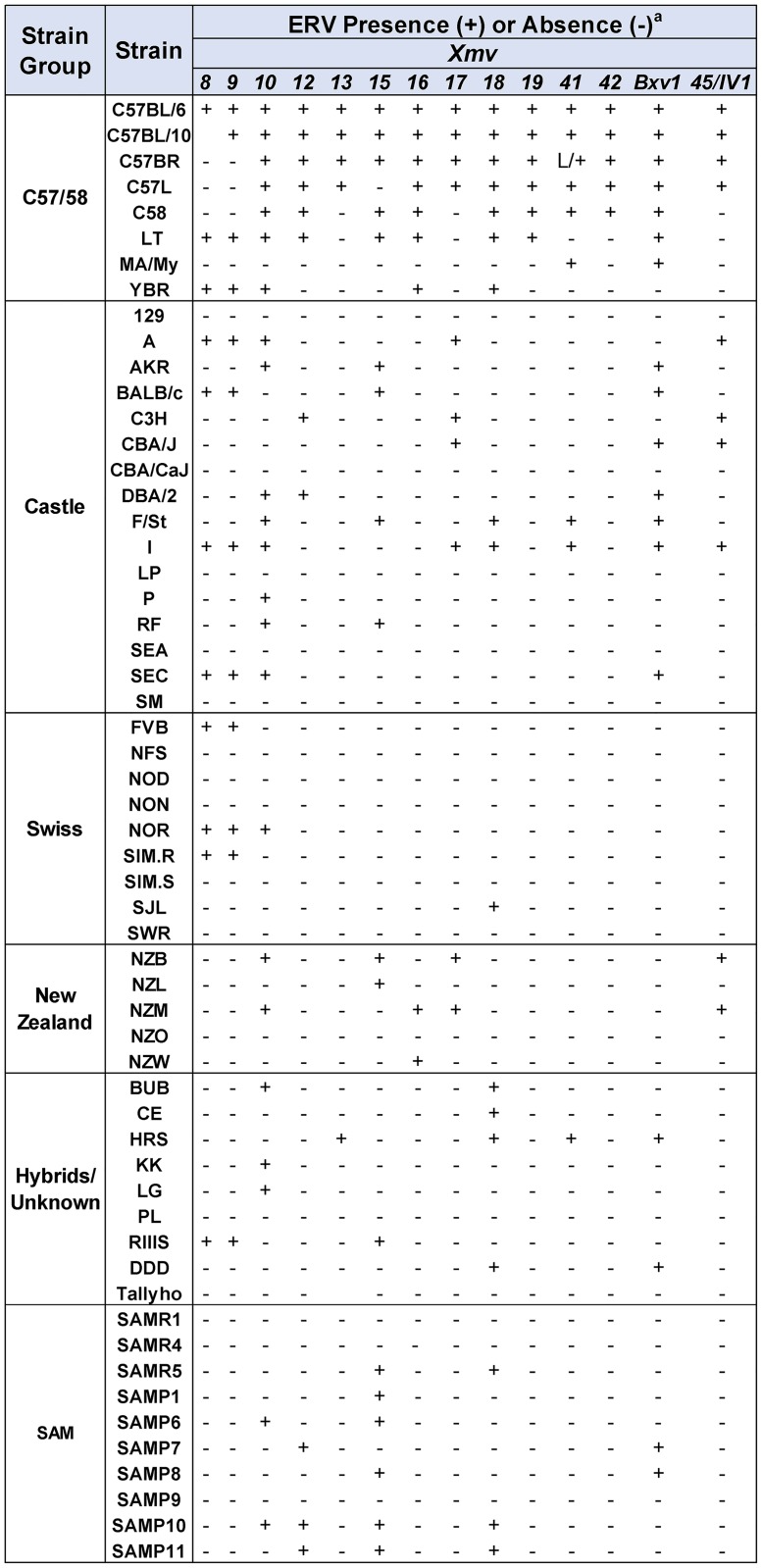
Classical mouse strains typed for 14 *Xmvs* and grouped according to known breeding history. ^a^+, presence; -, absence; L, solo LTR.

Our 58 mouse strain DNA panel (Figs 1 and 2) includes six sets of strains related by their origins and breeding histories: eight C57/58 strains that derive from a fancy mouse breeding set of three mice provided by hobbyist Abbie Lathrop to C.C. Little, 32 strains from colonies established by William Ernest Castle that also included breeders from Lathrop [14], nine Swiss mouse-derived strains, and nine unrelated strains derived from other, often poorly documented, sources [15]. The five New Zealand strains and ten strains of senescence accelerated mice (SAM) have been grouped with the Castle mice [15], but are discussed separately here.

**Fig 3:**
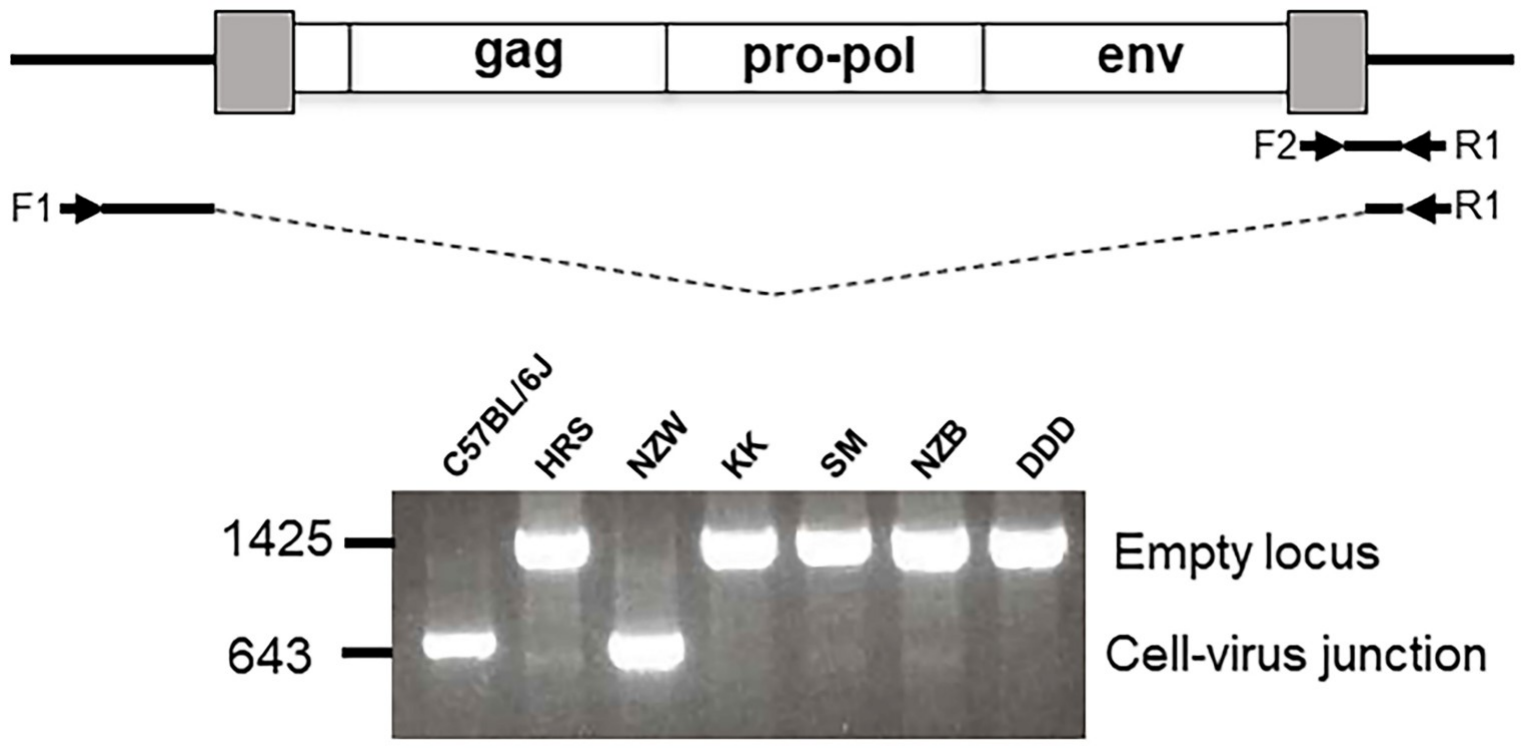
Detection of a representative ERV, *Pmv7*, in 7 inbred strains. At the top is a diagram of the provirus and cellular flanks with arrows showing positions of 3 primers and the expected products for mice with and without the *Pmv7* insertion. At the bottom are PCR test results for 7 strains using the three primers.

All mouse DNAs were screened by PCR for 45 X/P-ERVs present in the sequenced B6 genome [41] using ERV insertion-specific primer pairs [29]. These 45 ERVs include 14 *Xmvs*, 19 *Pmvs*, and 12 *Mpmvs*. Each DNA could be unambiguously typed for each ERV because, with a few exceptions described below, the primer sets for each ERV either produced expected cell-virus junction fragments or empty locus products (Fig 3 and S1 Fig). None of the 45 B6 ERVs is unique to B6, and each mouse strain carries a different subset of these ERVs (Figs 1 and 2).

The distribution of each ERV varies widely among the 58 strains. Although all strains carry at least six of the 31 P-ERVs, some individual ERVs are found in as few as three strains (*Mpmv5*) or in as many as 55 of the 57 strains (*Pmv1*, *Pmv10*) (Fig 1). Some of the distributional differences are lineage group-specific. Thus, three ERVs are restricted to the C57/58 strains (*Xmv19*, *Xmv42*, *Mpmv5*), and two (*Xmv12*, *Pmv5)*, were found only in the two sets of Lathrop-derived strains (C57/58, Castle). Ten ERVs were found in all six strain groups, including one *Xmv*, six *Pmvs*, and three *Mpmvs*, reflecting the intersubspecies mosaicism of inbred mice as well as possible cross-contamination due to inadvertent interbreeding [37]. 16 strains lack all 14 of the B6 *Xmvs*, and this absence is particularly notable in Swiss-derived strains, which is not surprising for mice derived from Western European stocks that lack X-MLVs [17].

**Fig 4:**
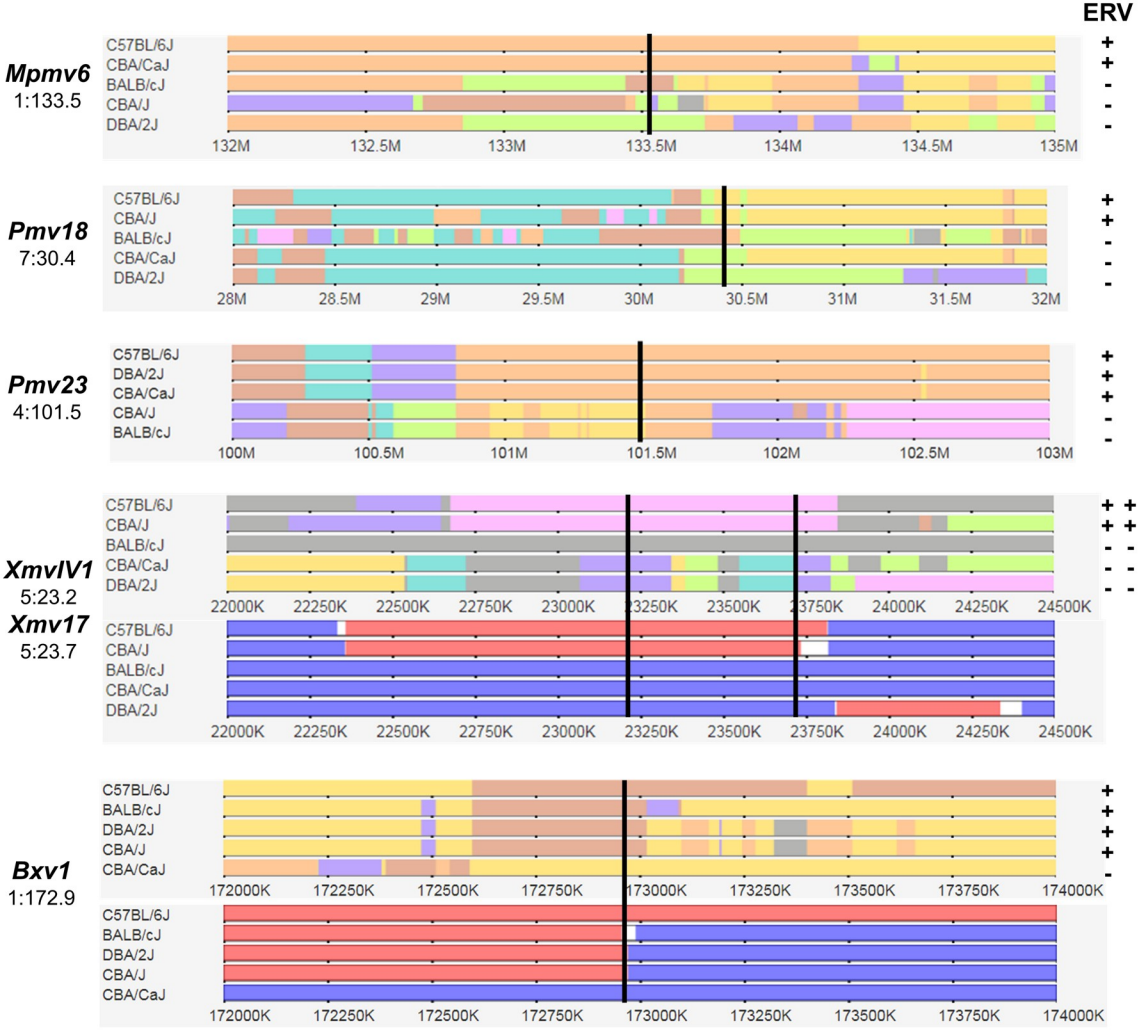
Wild mouse origins and haplotype identities for six ERVs. The horizontal tracks represent 2–4 megabase chromosome segments surrounding six specific ERV integration sites. ERVs and their genome locations are listed on the left and PCR typing data is on the right. The map locations for each ERV are marked by a vertical black line. Blocks of shared haplotypes are indicated in multiple colors; additional tracks for the three *Xmvs* show subspecies-specific blocks originating from *M*. *m*. *domesticus* in blue and from *M*. *m*. *musculus* in red.

All 14 *Xmvs* consistently map to MVP-defined genomic blocks in the sequenced mouse genome that are derived from *M*. *m*. *musculus* as shown previously for 13 *Xmvs* in a subset of these strains [29, 35]. All 31 P-ERV insertion sites map to segments of the mouse genome derived from *M*. *m*. *domesticus*. As shown for six representative ERVs in Fig 4, all P-ERVs also map to conserved haplotype blocks that distinguish the inbred strains, and all strains sharing ERV-linked haplotype blocks carry the relevant ERV. This consistent correlation of ERVs with subspecies and haplotype blocks shows that none are recent insertions and indicates that all predate the origins of laboratory mice. This resource should provide reliable predictions of ERV distributions in strains not typed by PCR that are included in the MPV dataset.

Inbred strains carry MLV ERVs not found in B6 [42, 43]. We searched the recently reported draft genome assemblies of 11 additional inbred strains [30] for MLV ERVs to support our PCR results and to identify ERVs that do not have B6 orthologs. All 11 of these sequenced and assembled strains (129, A, AKR, BALB/c, C3H/He, CBA, DBA/2, FVB, LP, NOD, NZO) were included in our PCR-typed panel. Not one full-length MLV ERV was identified in any of these 11 genomes despite the fact that eight of these strains are known to carry MLV ERVs capable of producing infectious virus constitutively or after induction (S1 Table). Southern blotting has identified specific *Emvs* in eight of the 11 genomes, four of which carry the same one, *Emv1*, and seven of these eight strains have been shown to produce infectious E-MLV (reviewed in [1]) (S1 Table). The virus-producing *Bxv1 Xmv* is carried by four of the newly assembled genomes. All of these previously mapped active ERVs were identified at the predicted sites in the relevant assemblies except for two of the three AKR *Emvs*, *Emv13* and *Emv14*. The previously unmapped *Emv* of LP mice, *Emv5*, [6] was positioned on Chr 9:17903062–17911495. All of these identified proviruses have substantial sequencing gaps and some also had duplications, insertions or rearrangements (S2 Fig). Screens of these assemblies for other *Pmvs*, *Mpmvs*, and *Xmv* also failed to identify any of the full-length or near full-length B6 ERVs determined to be present by PCR. Additional proviruses with no B6 orthologs were found in the 11 genomes, but all were also highly deleted, and their strain distributions could not be reliably determined. These results underscore the difficulty in reconstructing multicopy, insertionally polymorphic and sequence divergent ERVs in genome assemblies.

### Senescence-Accelerated Mouse (SAM) strains

The SAM strains originated from the inadvertent mating of AKR/J mice to mice of an unidentified strain or strains [24]. Because some of these animals showed early aging phenotypes, multiple inbred strains were developed from these mice, some of which were SAM-prone (SAMP) and some SAM-resistant (SAMR). The SAMP mice show a variety of early aging phenotypes such as activity loss, hair loss, and senile amyloidosis [44]. Average lifespans differ for SAMP (9.7 months) and SAMR (13.3 months) strains [45]. Like their AKR progenitor, SAM mice all carry *Emvs*, including the active AKR-derived *Emv11*, and most strains produce E-MLVs and some develop lymphomas [46]. E-MLVs have been linked to at least one aging phenotype, greying with age [47], but the individual *Emvs* in SAM strains cannot be linked to any specific aging phenotype [46].

We typed ten SAM strains by PCR for X/P-MLV ERVs (Figs 1 and 2). All 12 X/P-ERVs found in AKR were identified in one or more of the SAM strains, but seven ERVs not found in AKR were identified in the SAM strains (*Xmv12*,*18; Pmv1*,*18*,*19*,*23; Mpmv1*). The other progenitor(s) of the SAM strains have not been identified, but the only strain in our panel that carries all seven of these ERVs is B6. However, the fact that other gene mutations identified in SAMP strains are absent in C57BL [48] suggests that the unknown SAM progenitor is either a strain not included in the present analysis, or that there are multiple SAM progenitors.

### Solo LTRs (long terminal repeats)

Solo LTRs are generated by homologous recombination between ERV LTRs leading to excision of the intervening viral coding sequences. Such major deletions between terminal repeats were initially identified in the transposable elements found in yeast, *E*. *coli* and *Drosophila* [49–51], and were first described for MLVs in infected rat cells [52]. Solo LTRs derived from MLV ERVs were first detected for *Emv3* in DBA mice; this deletion was easily identified because it causes reversion of the dilute coat color mutation [53].

**Fig 5:**
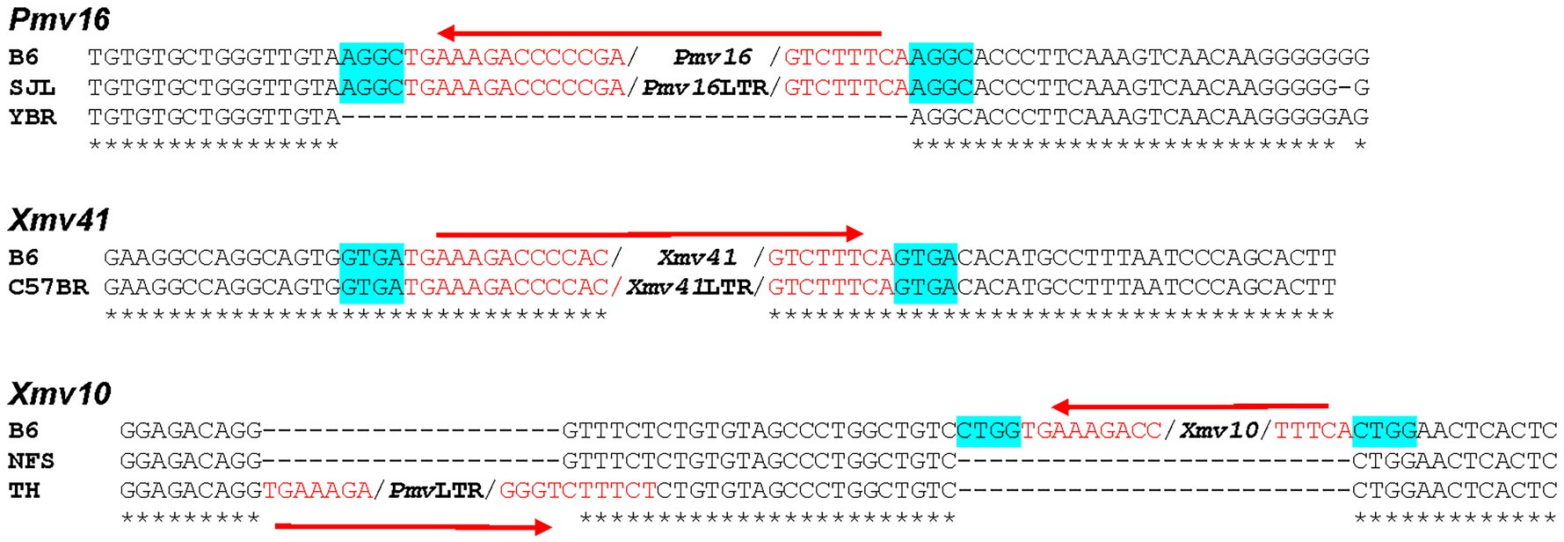
Solo LTRs at insertion sites of *Pmv16*, *Xmv41* and *Xmv10*. ERV sequences are in red. Flanking sequences of *Pmv16* and *Xmv41* show target site duplications, highlighted in blue, and these LTRs are at the same sites as the corresponding ERV in B6 mice. There is a *Pmv*-like solo LTR adjacent to the *Xmv10* site in TH mice; NFS is representative of strains that lack this LTR and *Xmv10*.

The number of solo LTRs relative to the number of full length viruses tends to be greater for more ancient ERVs suggesting progressive loss of coding sequences over time [43, 54, 55], but there is also evidence that solo LTRs are most frequently generated at or soon after endogenization and that such deletions decline with time as LTR sequences diverge [56]. In the course of this analysis, we identified five ERVs that produced empty locus amplicons that are ~500 bp larger than expected. Two of these larger amplicons have unrelated genomic inserts, but three contain solo LTRs (Fig 5), two of which correspond to the LTRs of the full-length ERV found at those sites in B6 mice.

*Pmv16* is found as a full-length provirus in B6, C57BL/10 and only one other strain, HRS (Figs 1 and 5). Six strains, such as YBR, carry the empty pre-integration site. The remaining 48 strains all carry a *Pmv16* solo LTR, shown for SJL. The prevalence of this deletion in all six strain groups indicates this solo LTR was acquired prior to the development of the inbred strains.

A second solo LTR, for *Xmv41*, was identified in only one strain, C57BR (Figs 2 and 5), but this deletion was found in only one of two C57BR DNAs tested, while the second sample produced the diagnostic PCR fragment for the empty locus. These two C57BR DNAs were prepared 30 years apart and otherwise showed the same typing results for other ERVs. The limited distribution of this solo LTR indicates that it is a recent, strain-specific deletion.

A third solo LTR was found using primers flanking the *Xmv10* insertion (Fig 5). This LTR was identified in two strains, MA/My and TH, strains that have no documented genealogical relationship [15]. 34 strains have the pre-integration site, shown for NFS (Figs 2 and 3). The sequence of this solo LTR, however, shows it is not derived from *Xmv10*, but is *Pmv*-like. This LTR is in reverse orientation relative to the *Xmv10* provirus, is inserted 19 bp from the *Xmv10* insertion site and overlaps the B6 reference sequence at its 3’ end accounting for the absence of a target site duplication (Fig 5). This solo LTR is thus a deletion of a *Pmv* not found in B6 and not preserved as a full-length provirus or solo LTR in any of the other strains.

### Disease links to specific ERVs and *Fv1* variants

Hundreds of individual inbred strains have been monitored for genetic and phenotypic variations including differences in strain lifespans, common genetic disorders and susceptibility to disease. We compiled the available data on the incidence of naturally occurring hematopoietic neoplasms, including T cell and non-T cell lymphomas as reported in the strain descriptions in the Mouse Genome Database [57] and in multiple studies and compilations that focused on specific strains or strain sets [23, 44, 58–65]. A 36 strain subset of our 58 strain panel had been typed for spontaneous lymphoma and also carry *Emvs*, expression of which is a necessary precondition for spontaneous lymphomagenesis. These 36 strains (Table 1) were grouped as high or as moderate-low disease incidence based on disease incidence, type and latency. Eight strains show a high incidence of T-cell lymphomas with early onset. 28 strains show low incidence or develop late onset neoplasms that are mostly B-cell, myeloid or reticular cell. The rest of our original 58 strain panel either do not carry *Emvs* and have low disease, or the incidence of hematopoietic neoplasms has not been reported.

**Table 1:** Characterization of inbred strains for naturally occurring hematopoietic neoplasms.

Disease Incidence, Latency, Type^a^	Strain	*Emvs*		X/P-ERVs Involved in P-MLV Generation^d^									*Fv1*
		*Locus*^b^	*Expression*^c^	*Pmvs*					*Xmvs*				
**High, Early, T Cell**	**AKR**	*Emv11-14*	SpH			*13*		*20*	*10*		*Bxv1*		***n***
	**C58**	4–6 *Emvs*	SpH	*1*	*11*	*13*		*20*	*10*		*Bxv1*		***n***
	**HRS**	*Emv1*,*3*	SpH	*1*	*11*	*13*	*15*	*20*			*Bxv1*		***n***
	**P**	*Emv3*	NT	*1*				*20*	*10*	*13*			***n***
	**PL**	3–4 *Emvs*	NT	*1*		*13*		*20*					***n***
	**SAMP7**	*Emv*11+1*Emv*	SpH	*1*		*13*					*Bxv1*		***n***
	**SAMP8**	*Emv*11+7*Emvs*	SpH	*1*		*13*					*Bxv1*		***n***
	**SAMP9**	*Emv*11+2*Emvs*	SpH	*1*		*13*		*20*					***n***
**Moderate, Late, Low, T Cell or Non-T Cell**	**C3H**	*Emv1*	Ind	*1*		*13*	*15*	*20*				*IV1*	***n***
	**C57BR**	*Emv2*	NT	*1*	*11*	*13*		*20*	*10*		*Bxv1*	*IV1*	***n***
	**CBA/J**	*Emv1*	Ag+	*1*		*13*	*15*	*20*		*13*		*IV1*	***n***
	**MA/My**	*Emv8*,*9*	(Ind),Ag+	*1*				*20*			*Bxv1*		***n***
	**NOD**	*Emv30*	SpL	*1*		*13*		*20*					***n***
	**SAMP1**	*Emv11*+2*Emvs*	SpH	*1*		*13*							***n***
	**SAMP10**	*Emv11*+5*Emvs*	SpH	*1*		*13*			*10*				***n***
	**SAMR5**	3*Emvs*	-	*1*		*13*		*20*					***n***
	**SEA**	*Emv1*,*3*	SpL,Ind	*1*	*11*	*13*		*20*					***n***
	**SJL**	*Emv9*,*10*	SpL,Ind,Ag+	*1*		*13*							***n***
	**NON**	*Emv30*	SpL	*1*		*13*		*20*					***n***
	**SAMP6**	2*Emvs*	-	*1*		*13*			*10*				***n***
	**SAMP11**	*Emv11*	SpH	*1*		*13*		*20*					***n***
	**SAMR1**	2*Emvs*	SpL	*1*		*13*		*20*					***n***
	**SAMR4**	2*Emvs*	SpL	*1*		*13*		*20*					***n***
	**F/St**	*Emv+*	SpH			*13*			*10*		*Bxv1*		***nr***
	**LP**	*Emv5*	NT	*1*	*11*	*13*							***nr***
	**KK**	*Emv+*	NT	*1*			*15*	*20*					***nr***
	**NZW**	*Emv+*	NT	*1*	*11*	*13*		*20*					***nr***
	**RF**	*Emv1*,*2*	Ag+	*1*		*13*		*20*	*10*				***nr***
	**SM**	*Emv1*	NT	*1*	*11*	*13*		*20*					***nr***
	**DBA/2**	*Emv3*	SpL	*1*		*13*		*20*	*10*		*Bxv1*		***d***
	**NZO**	*Emv+*	NT	*1*	*11*	*13*		*20*					***d***
	**A**	*Emv1*	Ind,Ag+	*1*		*13*		*20*	*10*			*IV1*	***b***
	**BALB/c**	*Emv1*	Ind,Ag+	*1*	*11*	*13*		*20*			*Bxv1*		***b***
	**C57BL**	*Emv2*	(Ind)	*1*	*11*	*13*	*15*	*20*	*10*		*Bxv1*	*IV1*	***b***
	**I**	*Emv1*,*3*	Ag-	*1*	*11*	*13*		*20*	*10*	*13*	*Bxv1*	*IV1*	***b***
	**YBR**	*Emv+*	NT	*1*	*11*	*13*		*20*	*10*				***b***

^a^Disease type and incidence was compiled from [23, 44, 57–65].

^b^Many *Emvs* have assigned numbers [6, 46]; *Emv*+, strains carrying uncharacterized PCR-detected *Emv env* sequences.

^c^Expression is identified as: SpH, spontaneous high; SpL, spontaneous low; Ag+, viral antigen positive; Ind, inducible by iododeoxyuridine; -, no expression; NT, not tested.

^d^The X/P-MLV ERVs that contribute to P-MLVs were previously identified [13].

**Fig 6:**
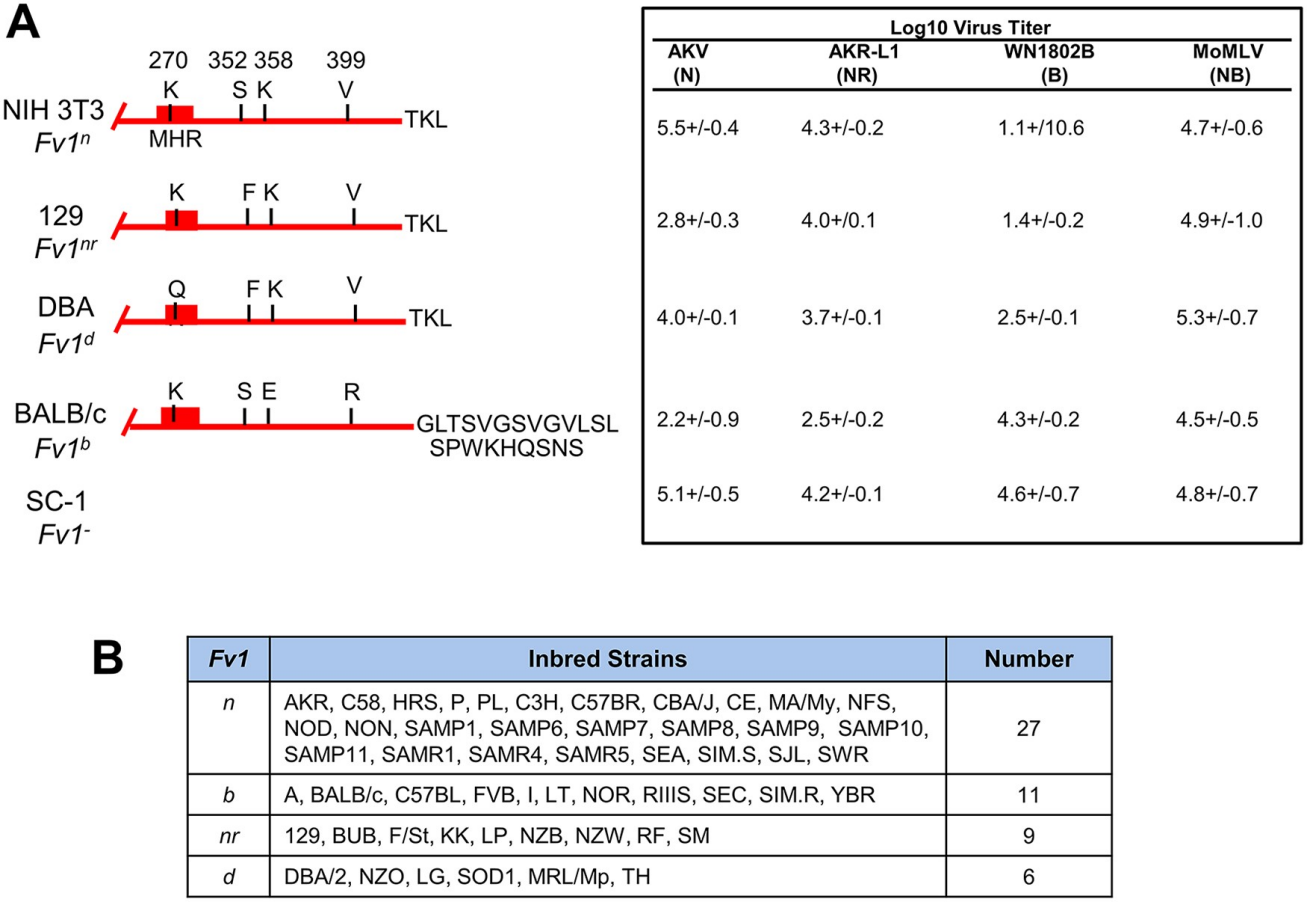
*Fv1* variants in classical inbred strains. A) The diagrams on the left identify protein sequence differences among the 4 inbred strain alleles. On the right are virus titers for E-MLVs sensitive to Fv1 restriction as determined for NIH3T3 (*Fv1*^*n*^), 129 (*Fv1*^*nr*^), DBA (*Fv1*^*d*^), BALB3T3 (*Fv1*^*b*^), and the wild mouse derived SC-1 (*Fv1*^-^). Log_10_ virus titers were determined by the UV-XC overlay assay; each cell line was tested 3–8 times. B) Inbred strains sequenced for *Fv1* alleles. Identical *Fv1*^*d*^ sequences were found for substrains DBA/2N and DBA/2J.

E-MLV viremia in mice that develop spontaneous lymphomas is followed by generation of P-MLVs having altered host range and enhanced virulence [13, 66, 67], and insertional mutagenesis by those P-MLVs. We examined the 36 strains for X/P-ERVs previously linked to P-MLV generation [13], and also typed them for variants of *Fv1*, a host gene that restricts replication of E- and P-MLVs and virus-induced disease [31, 68] (Table 1 and Fig 6).

#### *Fv1* allelic variation

There are four *Fv1* variants in inbred mice which restrict different subsets of mouse-tropic MLVs [69–71] (Fig 6A). E-MLVs from inbred strains are N-tropic, that is, not restricted by the *Fv1*^*n*^ allele. The *b*, *nr* and *d* alleles of *Fv1* restrict all or some N-tropic viruses (Fig 6A). PCR typing can identify *Fv1*^*b*^ due to a C-terminal 1.2kb insertion [72], but cannot distinguish the identically sized *Fv1*^*n*^, -*nr* and -*d* amplicons. Because only a handful of strains had been previously sequenced for *Fv1* alleles, we sequenced the *Fv1* genes in the strain subset typed for disease incidence and in additional strains chosen on the basis of their breeding histories. *Fv1*^*nr*^ [73] was found in a total of nine strains (Fig 6B), and *Fv1*^*d*^ [71] in six strains, including two strains not otherwise included in this study, SOD1/EiJ and MRL/MpJ. Our *Fv1*^*d*^ sequence, which differs from that in a previous report [74], resembles *Fv1*^*nr*^ in having the S352F substitution responsible for restricting some N-tropic viruses [75], but also contains a K270Q substitution, a site that is under strong positive selection [76] and has been linked to restriction of retroviruses other than MLVs [74]. Because only one *Fv1*^*b*^ gene, from B6, had been sequenced, we also sequenced the *Fv1* genes in five other strains typed by PCR as *Fv1*^*b*^ (A, BALB/c, I, LT, YBR) [77] (Fig 6B); all five had sequences identical to the *Fv1*^*b*^ B6 prototype.

Comparisons of *Fv1* allelic variation and lymphomagenesis showed that all 13 of the strains carrying one of the three restrictive *Fv1* alleles (*Fv1*^*b*^, ^*nr*^, ^*d*^), show moderate-low disease incidence, whereas all eight of the high leukemia strains carry *Fv1*^*n*^.

#### Active ERVs

The association between spontaneous lymphomagenesis and E-MLV production is clear for early onset thymomas, studied in strains like AKR or HRS. This association has also been demonstrated for late onset B-cell and myeloid leukemias through analysis of NFS.E-MLV+ congenics, AKXD RI strains and CFW Swiss mice carrying E-MLVs [78–80].

All 36 of the strains in Table 1 carry *Emvs*, but virus production patterns vary significantly. Six of the eight high leukemic strains have been typed for virus production and are all early, high producers. The majority of the low-moderate strains carry *Emvs* that show low, inducible and/or late virus production. A few moderate-low disease strains like SAMP1, 10, and 11, and F/St, however, show that a relatively high level of E-MLV expression is not sufficient for high disease incidence.

Infectious recombinant P-MLVs can potentially be generated in any mouse with replicating E-MLVs, but P-MLVs judged to be lymphomagenic by the AKR acceleration test [81], have only been isolated from the high leukemic strains [66]. Low disease incidence strains produce less complex P-MLV recombinants [12, 13] that are not lymphomagenic [66]. One factor that might explain the differences between pathogenic and nonpathogenic P-MLVs may be the strain differences in the complement of X/P-ERVs that contribute to the generation of these recombinants.

Our previous analysis of infectious P-MLVs identified segments acquired by recombination that showed homology to four *Xmvs* (*Bxv1*, *Xmv10*,*13*, *IV1)*, and five *Pmvs* (*Pmv1*,*11*,*13*,*15*,*20*) [13]. Six of these nine ERVs were implicated as likely progenitors of multiple independently isolated recombinant viruses suggesting these ERVs are especially active as recombination partners. The strain distribution of most of these ERVs in our mouse DNA panel is, however, not skewed toward high leukemic strains, with one exception (Table 1). *Bxv1* is an expressed X-ERV that is activated by immune stimulation [82]. *Bxv1* is present in only 25% of the low-moderate disease strains but is carried by 63% of the high strains. Most of the altered LTRs in pathogenic P-MLVs result from recombination with *Bxv1* [13] as also shown by restriction mapping and targeted sequencing of AKR mouse P-MLVs [83–85]. However, pathogenic LTRs can also be produced by mutation in mice that lack *Bxv1* [13, 86], indicating that *Bxv1* is important but not necessary for the generation of disease-inducing viruses.

The failure to identify specific P-ERVs linked to disease is likely due to several factors. First, late-expressing *Emvs* may not provide enough time for the necessary multiple rounds of recombination needed to produce complex lymphomagenic recombinants. Second, acquisition of *Pmv env* sequences is necessary for P-MLV host range, but is not sufficient for the generation of pathogenic P-MLVs, and the segment of the TM*env* linked to lymphomagenesis [13] can derive from multiple P-ERVs. Third, inbred strains carry P-ERVs not found in the reference low-incidence B6 mouse genome [42, 43]. Such copies in the high disease strains but not in B6 may be more likely to produce pathogenic P-MLVs, but their identification awaits the completed sequencing of multiple classical strains and the accurate annotation of their ERV content.

## Conclusions

Here we characterized 58 strains to describe the distributional patterns of 45 MLV ERVs that can be present, absent or have undergone deletion to produce solo LTRs, and for functional variants of the host *Fv1* gene that inhibit MLV spread. This study provides insight into the host factors responsible for strain differences in naturally occurring virus-induced diseases and helps characterize the ERV content of the various classical inbred strain genomes. The distribution of individual ERVs often reflects common strain ancestry, and we identified several ERVs alternatively present as full-length MLVs or deleted solo LTRs. This dataset adds to our understanding of strain relationships and the disease implications of ERV content, and should assist in annotating genomic sequences of strains that differ from the framework B6 sequence in ERV content. While first draft sequencing of additional mouse strains has been completed, the incomplete annotation of their MLV ERV content illustrates the difficulty in reconstructing the sequences of repetitive and insertionally polymorphic ERVs. As shown here and previously [87], even ERVs capable of producing infectious virus have significant sequencing gaps in newly produced assemblies.

Classical inbred strains have long been used to identify genetic differences controlling susceptibility to lifespan-shortening diseases. Our data identified several patterns related to the inheritance and expression of functional MLV ERVs. First, restrictive alleles of *Fv1* are linked to lower leukemia incidence. Second, highly expressed *Emvs* are necessary but not sufficient for early lymphomagenesis. Third, the nonecotropic B6 ERVs previously identified as likely progenitors of pathogenic recombinants are not overrepresented in the high leukemia strains, with the exception of *Bxv1* which is a common contributor to the generation of pathogenic viruses [13, 85]. This suggests that high leukemia strains may carry additional P-ERVs that can help create pathogenic recombinants.

## Supporting information

S1 Fig Uncropped PCR gel used for Fig 3. Lane 1, markers (Invitrogen Trackit 1Kb Plus DNA Ladder, Cat No. 10488085); Lanes 2–8, products of an unrelated PCR; Lanes 9–15, empty locus and cell-virus junction fragments for *Pmv7*.
(PPTX)

S2 Fig Proviral sequences of known *Emvs* and *Bxv1* orthologs in 9 inbred strain assemblies. At the top is a diagram of the MLV proviral genome identifying locations of the LTRs with U3-R-U5 structures and *gag*, *pol* and *env* genes. Proviruses are identified on the right; *Emv1* and *Bxv1* are each present in multiple strains. Thick lines are viral sequences. Dotted lines are sequencing gaps. Red lines mark duplications. An inserted sequence is in green. Blue lines are misplaced segments with assembly positions shown by arrows. Brown lines are unplaced sequences.
(PPTX)

S1 Table *Emvs* and *Bxv1* orthologs in assembled genomes of inbred mouse strains. Listed ERVs are constitutively expressed or inducible and are found in eight of the 11 genomes [88–98]. Three assembled genomes (129, FVB, NZO) have no full-length MLV ERVs capable of producing virus. *Emv1-3* have small defects correctable by mutation or recombination.
(XLSX)
