# Supplementary figures and images for: Distribution of endogenous gammaretroviruses and variants of the Fv1 restriction gene in individual mouse strains and strain subgroups
Source: PLoS One. 2019 Jul 10;14(7):e0219576. doi: 10.1371/journal.pone.0219576 (PMC6619830; doi:10.1371/journal.pone.0219576)

## Slide 1
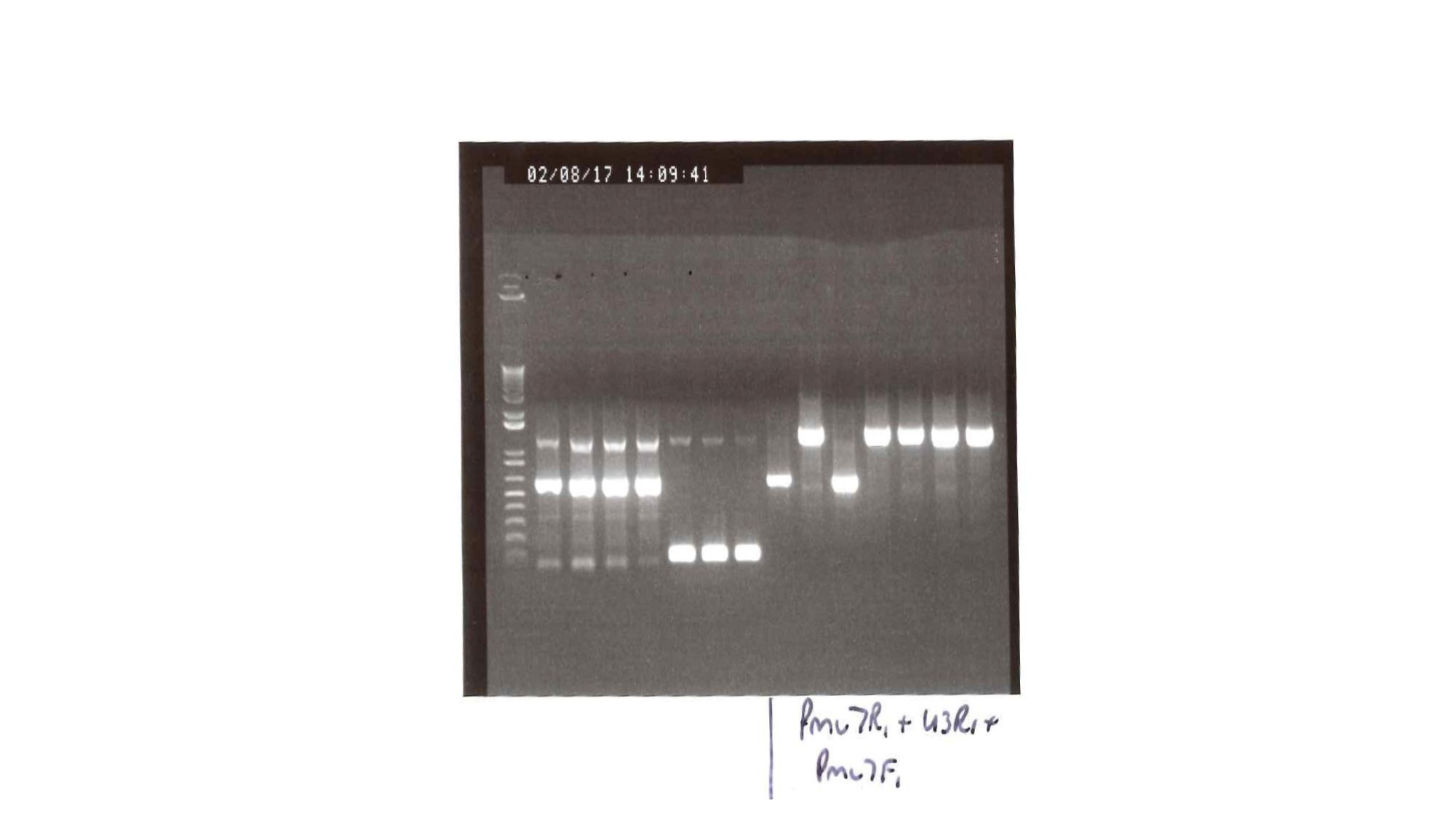

Supplement: S1 Fig — Lane 1, markers (Invitrogen Trackit 1Kb Plus DNA Ladder, Cat No. 10488085); Lanes 2–8, products of an unrelated PCR; Lanes 9–15, empty locus and cell-virus junction fragments for Pmv7. (PPTX) [file pone.0219576.s001.pptx]
