# Supplementary material for: Distribution of endogenous gammaretroviruses and variants of the Fv1 restriction gene in individual mouse strains and strain subgroups
Source: PLoS One. 2019 Jul 10;14(7):e0219576. doi: 10.1371/journal.pone.0219576 (PMC6619830; doi:10.1371/journal.pone.0219576)
Supplement: S2 Fig — At the top is a diagram of the MLV proviral genome identifying locations of the LTRs with U3-R-U5 structures and gag, pol and env genes. Proviruses are identified on the right; Emv1 and Bxv1 are each present in multiple strains. Thick lines are viral sequences. Dotted lines are sequencing gaps. Red lines mark duplications. An inserted sequence is in green. Blue lines are misplaced segments with assembly positions shown by arrows. Brown lines are unplaced sequences. (PPTX) [file pone.0219576.s002.pptx]

## Slide 1
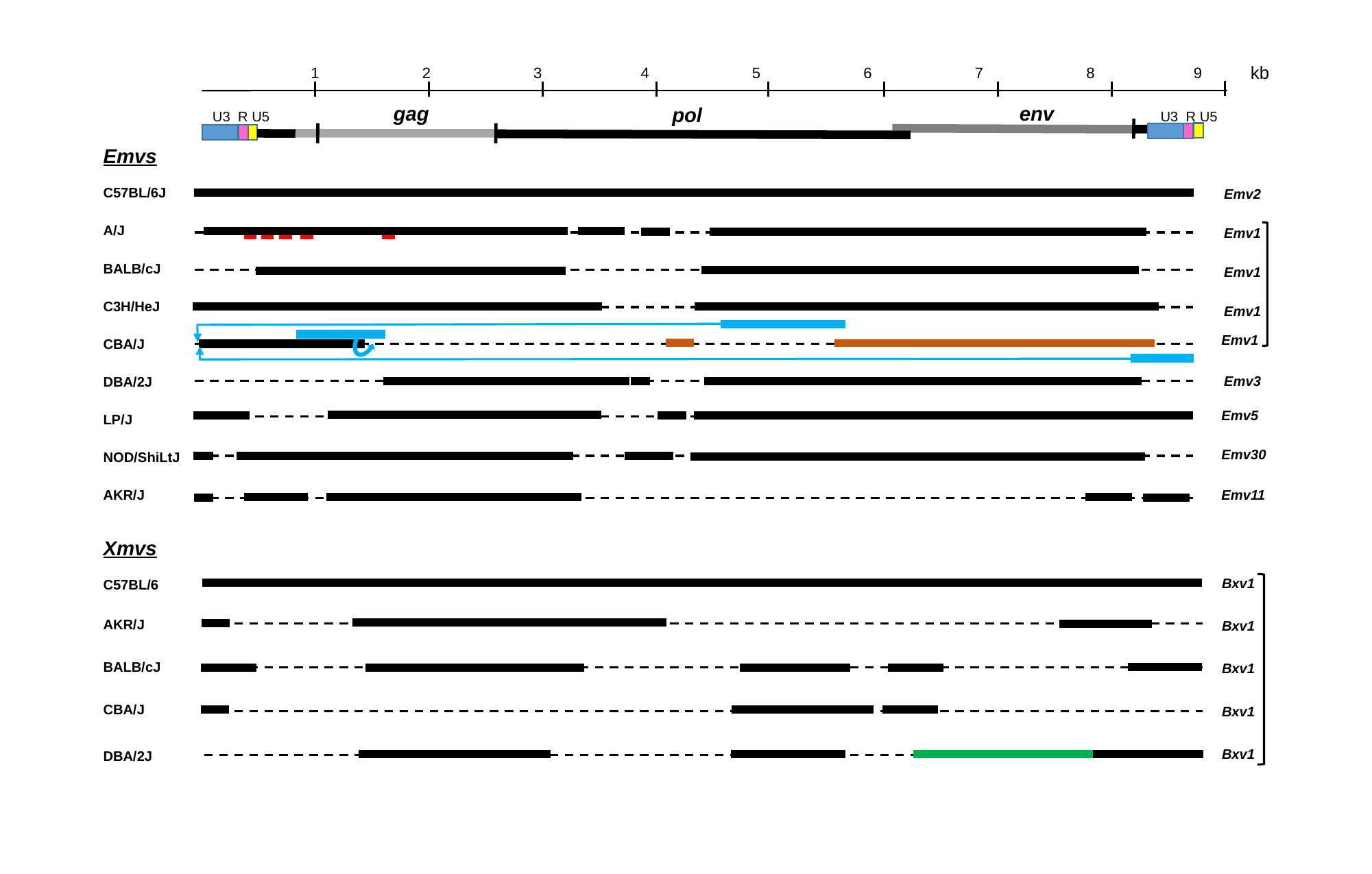

kb
1 2 3 4 5 6 7 8 9
env
gag
pol
 U3 R U5
U3 R U5
Emvs
C57BL/6J
Emv2
A/J
Emv1
BALB/cJ
Emv1
C3H/HeJ
Emv1
Emv1
CBA/J
Emv3
DBA/2J
Emv5
LP/J
Emv30
NOD/ShiLtJ
AKR/J
Emv11
Xmvs
Bxv1
C57BL/6
AKR/J
Bxv1
BALB/cJ
Bxv1
CBA/J
Bxv1
Bxv1
DBA/2J
